# Cannabis Usage Among Patients With Hidradenitis Suppurativa: A Scoping Review

**DOI:** 10.1177/12034754241238719

**Published:** 2024-03-10

**Authors:** Dea Metko, Shanti Mehta, Vincent Piguet

**Affiliations:** 1 Michael G. DeGroote School of Medicine, McMaster University, Hamilton, ON, Canada; 2 Temerty Faculty of Medicine, University of Toronto, Toronto, ON, Canada; 3 Division of Dermatology, Department of Medicine, Temerty Faculty of Medicine, University of Toronto, Toronto, ON, Canada; 4 Division of Dermatology, Department of Medicine, Women’s College Hospital, Toronto, ON, Canada

**Keywords:** hidradenitis suppurativa, acne inversa, cannabis, marijuana, chronic pain

To the Editor,

Hidradenitis suppurativa (HS) is a chronic inflammatory skin condition characterized by the formation of painful nodules, abscesses, and deep scarring typically in flexural areas. Patients often report severe pain and acute discomfort especially during flares, thus making pain relief a vital component in the management of HS. We set out to explore previous literature regarding cannabis usage among HS patients to investigate the potential role of cannabis in the pain management and outcomes of HS.

A search of OVID’s Medline and Embase databases was completed from inception on December 4, 2023, according to the Preferred Reporting Items for Systematic Reviews and Meta-Analyses Extension for Scoping Reviews (PRISMA-ScR). A summary of the findings from the included literature can be found in Supplemental Table S1. The full search strategy is provided in Supplemental Table S2, as well as the articles included (Supplemental Figure S1).

When exploring methods of pain relief, marijuana, both in smoking and edible forms, was identified as the most effective therapy for pain management. Marijuana was reported to be more effective than the well-known pain modulators such as acetaminophen and had slightly greater rates of patient-reported relief than opioids.^1^

A distinctive cannabis usage trend was noted among HS patients in comparison to other uncomfortable chronic skin conditions (ie, psoriasis), with increased quantity, frequency, and proportion of use. Recreation was the primary motivator for use, while patients with more severe HS manifestations cited pain, stress, and moral support as additional motivations. Interestingly, no significant difference was found in Hurley stage, or dermatology quality of life index scores between cannabis users and nonusers.^2^

A survey of 210 patients with dermatological conditions, including HS, revealed a substantial proportion of the cohort engaged with substance use, although only a fraction attributed this behaviour specifically toward their skin conditions. Cannabis/cannabinoid creams/oils were favoured, with a majority reporting improvement in their skin condition.^3^ However, patients with HS also exhibited significantly heightened odds of substance abuse disorder (SUD) across demographic subgroups, with a particularly pronounced association in those aged 45 to 64 years, patients with skin of color, and those without concurrent depressive or anxiety disorders.^4^

Another study carried out a comprehensive analysis of online posts on a HS dedicated discussion page on Reddit, revealing HS patients regard cannabis as a focal point of interest in pain relief. Cannabis usage was mentioned in contexts of pain management, mental health, and clinical presentation in this online community.^5^

This scoping review provides a summation of evidence suggesting that cannabis is widely used by HS patients and might provide benefits for managing pain. It also illuminates irregular usage patterns and inclination toward SUD in this patient population. Limitations include a small number of studies reporting cannabis use in HS patients, and a lack of description of specific cannabinoid products used (Tetrahydrocannabinol (THC), cannabidiol (CBD)). Further research is crucial to determine the pattern of usage and the potential role of cannabis-derived products in managing patients with HS.

## Supplemental Material

sj-docx-1-cms-10.1177_12034754241238719 – Supplemental material for Cannabis Usage Among Patients With Hidradenitis Suppurativa: A Scoping Review Supplemental material, sj-docx-1-cms-10.1177_12034754241238719 for Cannabis Usage Among Patients With Hidradenitis Suppurativa: A Scoping Review by Dea Metko, Shanti Mehta and Vincent Piguet in Journal of Cutaneous Medicine and Surgery
