# Supplementary material for: Cannabis Usage Among Patients With Hidradenitis Suppurativa: A Scoping Review
Source: J Cutan Med Surg. 2024 Mar 10;28(3):307–8. doi: 10.1177/12034754241238719 (PMC11141092; doi:10.1177/12034754241238719)
Supplement: sj-docx-1-cms-10.1177_12034754241238719 – Supplemental material for Cannabis Usage Among Patients With Hidradenitis Suppurativa: A Scoping Review [file sj-docx-1-cms-10.1177_12034754241238719.docx]

**Supplemental Material for:**

**Cannabis Usage Amongst Patients with Hidradenitis Suppurativa: A Scoping Review**

**Supplementary Figure 1.** Flow diagram of literature screening using the Preferred Reporting Items for Systematic reviews and Meta-Analyses extension for Scoping Reviews (PRISMA-ScR)

^
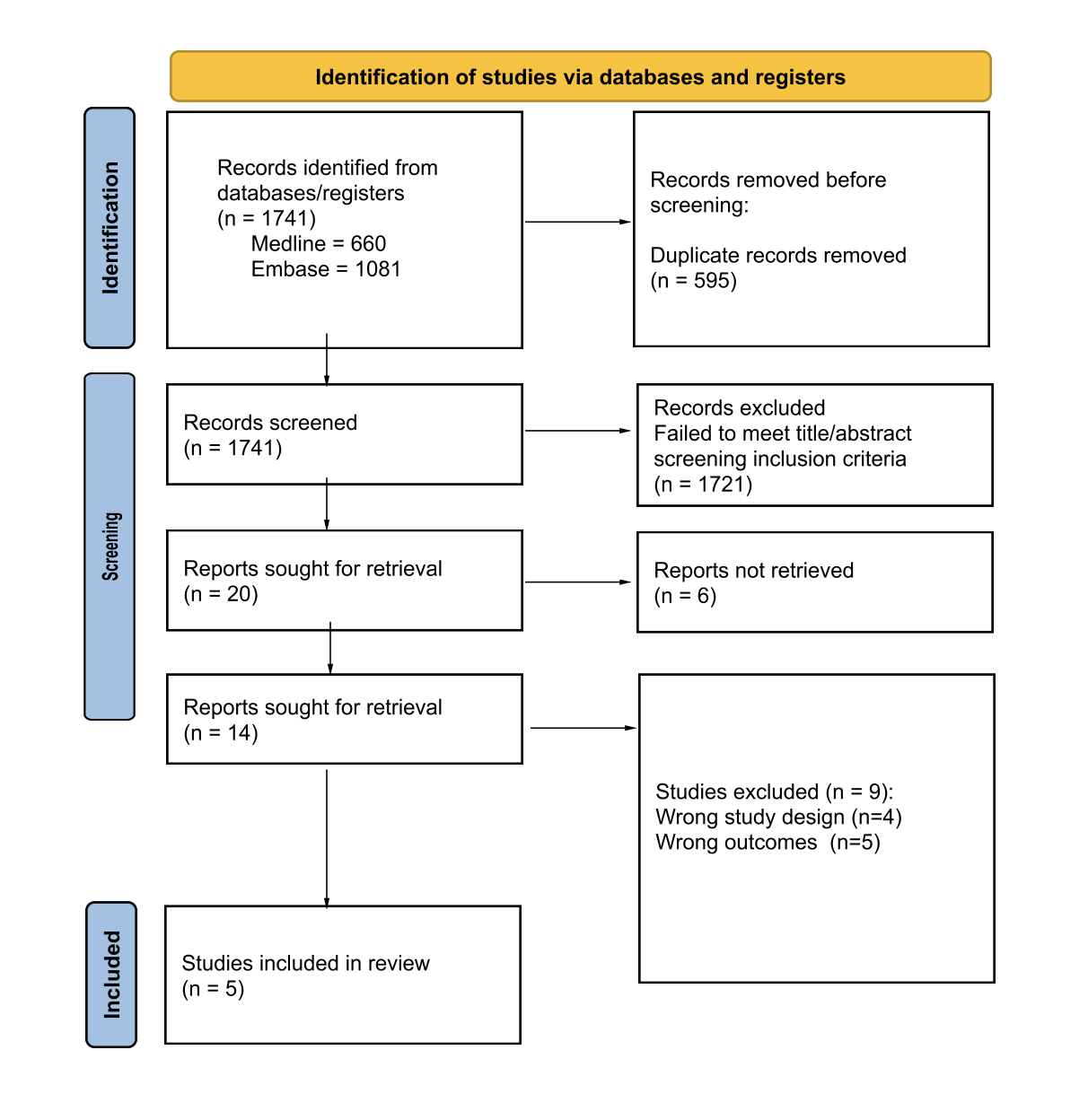
^

**Supplementary Table 1.** Summary of the screened literature.

| **Author, year of publication** | **Aim of study** | **Sample size** | **Methods** | **Results** |
| --- | --- | --- | --- | --- |
| Fernandez, 2022 | To identify pain management therapies used in HS and their perceived effectiveness | 438 | Survey | Marijuana smoking received the highest mean effectiveness rating (2.92 ± 1.10) for pain management, followed by marijuana edibles (2.87 ± 1.10). Marijuana was found to be more effective than ibuprofen (2.02, p < .0001) and acetaminophen (1.71, p < .0001). |
| Garg, 2018 | To evaluate substance use disorder among HS patients in the United States | 32,625 | Cross-sectional analysis | Cannabis accounted for 29.7% of substance use abuse among HS patients, and patients with HS. had 1.50 (95% confidence interval 1.42-1.59) times the adjusted odds of SUD compared to patients without HS. |
| Lesort, 2019 | To determine the prevalence and reason for cannabis use in HS patients | 641 | Multicentric prospective case–control study | The prevalence of cannabis use during the past 12 months among the HS group was 34.0% [95% confidence interval (CI) 30–38]. Patients with more severe disease, assessed by a higher DLQI score, more often reported pain as a motivation for cannabis use (P < 0·001), but also stress (P = 0·010) and moral support (P < 0·001). |
| Mahurin, 2020 | To investigate the prevalence and form of cannabis use among dermatology patients | 210 | Survey | 38.2% of respondents reported using cannabis or cannabinoid products, but only 10.6% of respondents had used these to treat their skin condition. Most of these patients reported at least some improvement in their skin condition with cannabis/ cannabinoid use. |
| Rajalinga, 2022 | To investigate HS-related patient concerns on online forum posts | 6,948 | Retrospective database analysis | Of 61,627 posts made by 6948 users on an HS reddit forum, 1.1% were on the topic of cannabis |

HS, Hidradenitis suppurativa; DLQI, Dermatology Life Quality Index

**Supplementary Table 2.** Search strategy.
Database:

**Embase** <1974 to 2023 December 04>

| **#** | **Query** | **Results from 5 Dec 2023** |
| --- | --- | --- |
| 1 | exp hidradenitis axillaris/ or hidradenitis suppurativa.mp. or acne inversa.mp. or apocrine acne.mp. or apocrinitis.mp. or Fox-den disease.mp. or pyoderma sinifica fistulans.mp. or Velpeau's disease.mp. or Verneuil's disease.mp. | 6,865 |
| 2 | exp cannabis/ or weed.mp. or marijuana.mp. | 67,478 |
| 3 | exp pain/ or pain management.mp. or pain*.mp. | 2,209,233 |
| 4 | 2 or 3 | 2,269,945 |
| 5 | 1 and 4 | 1,670 |

**Ovid MEDLINE(R) and Epub Ahead of Print, In-Process, In-Data-Review & Other Non-Indexed Citations, Daily and Versions** <1946 to December 04, 2023>

| **#** | **Query** | **Results from 5 Dec 2023** |
| --- | --- | --- |
| 1 | exp hidradenitis axillaris/ or hidradenitis suppurativa.mp. or acne inversa.mp. or apocrine acne.mp. or apocrinitis.mp. or Fox-den disease.mp. or pyoderma sinifica fistulans.mp. or Velpeau's disease.mp. or Verneuil's disease.mp. | 4,634 |
| 2 | exp cannabis/ or weed.mp. or marijuana.mp. | 42,019 |
| 3 | exp pain/ or pain management.mp. or pain*.mp. | 1,055,745 |
| 4 | 2 or 3 | 1,095,356 |
| 5 | 1 and 4 | 666 |
